# Schwannoma Likely Arising from the Pancreatic Head Nerve Plexus That Mimicked a Mucinous Cystic Neoplasm and Was Treated by Pancreaticoduodenectomy: A Case Report

**DOI:** 10.70352/scrj.cr.26-0488

**Published:** 2026-09-04

**Authors:** Shingo Kozono, Hirokuni Hazama, Yasuna Manabe, Chiaki Nakahara, Norimasa Abe, Atsushi Fujii, Keisuke Hirahata, Yuzo Shimokawa, Masayuki Hijioka, Hirotaka Kuga, Masato Sakamoto, Sadafumi Tamiya, Toru Nakano

**Affiliations:** 1 Department of Surgery, Kitakyushu Municipal Medical Center, Kitakyushu, Fukuoka, Japan; 2 Department of Pathology, Kitakyushu Municipal Medical Center, Kitakyushu, Fukuoka, Japan; 3 Department of Gastroenterology, Kitakyushu Municipal Medical Center, Kitakyushu, Fukuoka, Japan; 4 Department of Cardiovascular Surgery, Kitakyushu Municipal Medical Center, Kitakyushu, Fukuoka, Japan

**Keywords:** pancreatic schwannoma, peripancreatic schwannoma, pancreatic head nerve plexus, mucinous cystic neoplasm, pancreaticoduodenectomy, arterial reconstruction, replaced right hepatic artery

## Abstract

**INTRODUCTION:**

Pancreatic and peripancreatic schwannomas are exceptionally rare benign peripheral nerve sheath tumors. Cystic and hemorrhagic degeneration may produce imaging findings resembling pancreatic cystic neoplasms, while an extrapancreatic neural origin can be difficult to recognize preoperatively. Lesions located in the pancreatic head nerve plexus region pose additional surgical challenges because of their close relationship with periarterial nerve plexuses and major vessels.

**CASE PRESENTATION:**

A 48-year-old woman was referred for a 5-cm multilocular cystic lesion adjacent to the pancreatic head. CT, MRI, and endoscopic ultrasonography showed a thick-walled multilocular cystic mass with enhancing septa and solid components, hemorrhagic change, no communication with the main pancreatic duct, and marked displacement of peripancreatic vessels. An MCN-like cystic neoplasm with malignant potential was suspected. Endoscopic US-guided tissue acquisition was not performed because puncture-related dissemination was a concern. Pancreaticoduodenectomy was selected because malignancy could not be excluded and safe local excision was considered technically difficult. Intraoperatively, the tumor was well demarcated from the pancreatic parenchyma but firmly adherent to the periarterial nerve plexus around the common hepatic artery, celiac axis, splenic artery, and superior mesenteric artery. A replaced right hepatic artery was in broad contact with the tumor and was resected en bloc with the tumor and reconstructed. The tumor was removed without capsular rupture. Histopathological examination showed a spindle cell neoplasm with cystic and hemorrhagic degeneration located in the pancreatic head nerve plexus region, with the surrounding nerve plexus tissue around the tumor and no epithelial lining. Immunohistochemistry showed diffuse positivity for S-100 protein and SRY-box transcription factor 10 (SOX10) and negativity for c-kit, discovered on gastrointestinal stromal tumor 1 (DOG1) CD34, α-SMA, desmin, and pan-cytokeratin AE1/AE3 (AE1/AE3), supporting the diagnosis of schwannoma likely arising from the pancreatic head nerve plexus. The postoperative course was uneventful.

**CONCLUSIONS:**

A schwannoma located in the pancreatic head nerve plexus region may mimic an MCN-like cystic neoplasm and make periarterial dissection technically demanding. When clinically appropriate and safely feasible, EUS-guided tissue acquisition may be considered because a preoperative diagnosis may permit observation or organ-preserving surgery. Pancreaticoduodenectomy may be selected when malignancy cannot be excluded and safe local excision is not feasible because of firm periarterial adhesion.

## Abbreviations

ADC
: apparent diffusion coefficient
CA19-9
: carbohydrate antigen 19-9
CBD
: common bile duct
CEA
: carcinoembryonic antigen
CHA
: common hepatic artery
EGFR
: epidermal growth factor receptor
EUS
: endoscopic ultrasonography
EUS-FNA
: endoscopic US-guided fine-needle aspiration
EUS-FNB
: endoscopic US-guided fine-needle biopsy
FDG-PET/CT
: fluorodeoxyglucose-PET/CT
GIST
: gastrointestinal stromal tumor
IPMN
: intraductal papillary mucinous neoplasm
LHA
: left hepatic artery
MCN
: mucinous cystic neoplasm
MRCP
: magnetic resonance cholangiopancreatography
RHA
: right hepatic artery
SA
: splenic artery
SMA
: superior mesenteric artery
SMV
: superior mesenteric vein
α-SMA
: alpha-smooth muscle actin

## INTRODUCTION

A schwannoma is a benign peripheral nerve sheath tumor, but its occurrence in the pancreatic or peripancreatic region is exceptionally rare. Preoperative diagnosis can be difficult because schwannomas may undergo cystic degeneration, hemorrhage, and hyalinization, producing a mixed solid and cystic or multilocular cystic appearance. Consequently, pancreatic schwannomas can closely mimic more common pancreatic cystic neoplasms, including MCN, serous cystic neoplasm, and solid-pseudopapillary neoplasm.^1–3)^

A schwannoma located in the pancreatic head nerve plexus region presents an additional diagnostic and surgical challenge. Peripancreatic schwannomas may arise from extrapancreatic nerve plexuses adjacent to the pancreas, including the pancreatic head nerve plexus and the superior mesenteric, celiac, and splenic plexuses.^4,5)^ Because these tumors are anatomically close to the pancreatic parenchyma and major peripancreatic vessels, their origin may be difficult to determine preoperatively, and they may be radiologically interpreted as primary pancreatic tumors.^4)^ This distinction is clinically important because schwannomas are generally benign, and observation or organ-preserving surgery may be appropriate when a confident diagnosis is established before treatment.^4,6)^ However, this principle may be difficult to apply when the tumor is located in the pancreatic head nerve plexus region, where diagnostic uncertainty and close anatomical relationships with the periarterial nerve plexus and major vessels can substantially influence surgical planning.

We herein report a case of a schwannoma likely arising from the pancreatic head nerve plexus that mimicked an MCN-like cystic neoplasm and was treated by pancreaticoduodenectomy with resection and reconstruction of a replaced RHA. This case highlights the importance of a broad preoperative differential diagnosis, the potential role of EUS-guided tissue acquisition, and the need to individualize the extent of surgery according to diagnostic uncertainty regarding malignancy and intraoperative anatomy.

## CASE PRESENTATION

A 48-year-old woman was referred to our department for evaluation of a cystic lesion in the pancreatic head region. She had experienced intermittent epigastric pain for 4–5 years. CT performed at a local hospital revealed a cystic pancreatic mass, and she was referred to our institution for further evaluation and treatment. She had no relevant family history, took no regular medications, and had no history of smoking or alcohol consumption. Physical examination revealed no palpable abdominal mass or abdominal tenderness. Laboratory testing showed mild anemia, with a hemoglobin level of 9.1 g/dL. Serum CEA and CA19-9 levels were within the normal ranges at 1.8 ng/mL and 26.7 U/mL, respectively.

**Fig. 1:**
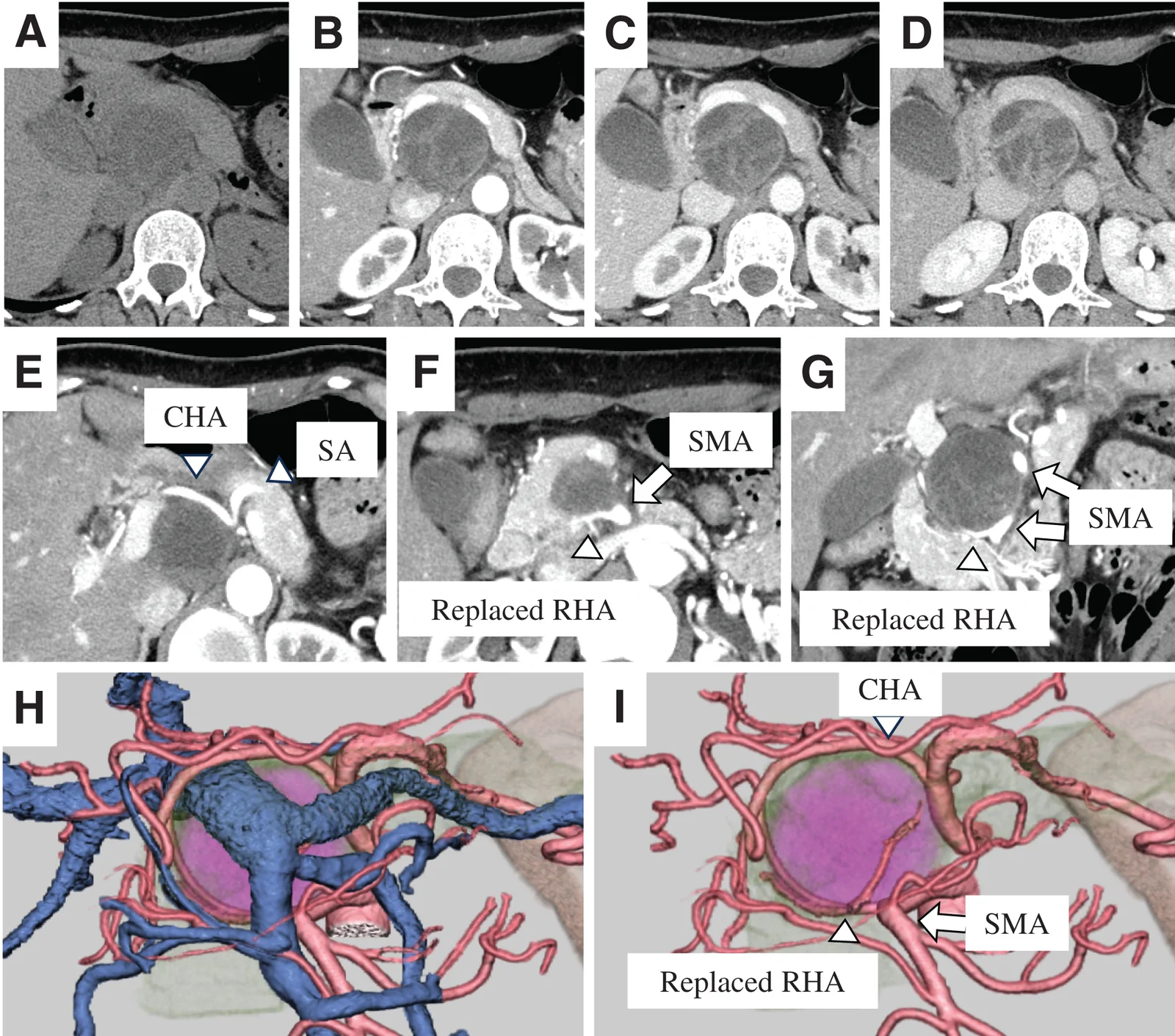
Contrast-enhanced CT and 3D vascular reconstruction findings. (**A**–**D**) Axial CT images obtained during the unenhanced (**A**), arterial (**B**), portal venous (**C**), and equilibrium (**D**) phases showing an approximately 5-cm, well-demarcated multilocular cystic mass adjacent to the pancreatic head. The lesion compresses the pancreatic parenchyma and the porto-mesenteric venous axis anteriorly and contains internal septa and focally thickened areas showing gradual enhancement across the dynamic phases. (**E**) Axial contrast-enhanced image showing marked displacement and compression of the CHA by the tumor. (**F**) Axial contrast-enhanced image showing displacement and compression of the SMA and its branch, the replaced RHA, by the tumor. (**G**) Coronal reconstructed image demonstrating the same findings as in (**F**), showing displacement and compression of the SMA and the replaced RHA by the tumor. (**H**, **I**) 3D vascular reconstruction images demonstrating the close anatomical relationship between the tumor and the peripancreatic vessels, including the CHA, SMA, replaced RHA, and porto-mesenteric venous axis. CHA, common hepatic artery; RHA, right hepatic artery; SA, splenic artery; SMA, superior mesenteric artery

Contrast-enhanced CT demonstrated an approximately 5-cm, well-demarcated multilocular cystic mass adjacent to the pancreatic head (**Fig. 1A**–**1D**). The lesion compressed the pancreatic parenchyma and porto-mesenteric venous system anteriorly and contained internal septa and focally thickened areas that showed gradual enhancement across the dynamic phases. These findings, particularly the multilocular cystic architecture, enhancing septa, and focally thickened enhancing areas suggestive of mural nodules or solid components, raised suspicion of an MCN-like cystic neoplasm with malignant potential. No lymph node, liver, or lung metastases were identified. The tumor caused marked displacement and compression of the peripancreatic vessels, including the CHA, SMA, and replaced RHA (**Fig. 1E**–**1G**). Although the replaced RHA was in broad contact with the tumor, preoperative imaging showed only displacement and compression, without definite encasement, luminal narrowing, or contour irregularity suggestive of arterial invasion. 3D vascular reconstruction further delineated the close anatomical relationship between the tumor and the surrounding vasculature, including the CHA, SMA, replaced RHA, and porto-mesenteric venous axis (**Fig. 1H**, **1I**).

**Fig. 2:**
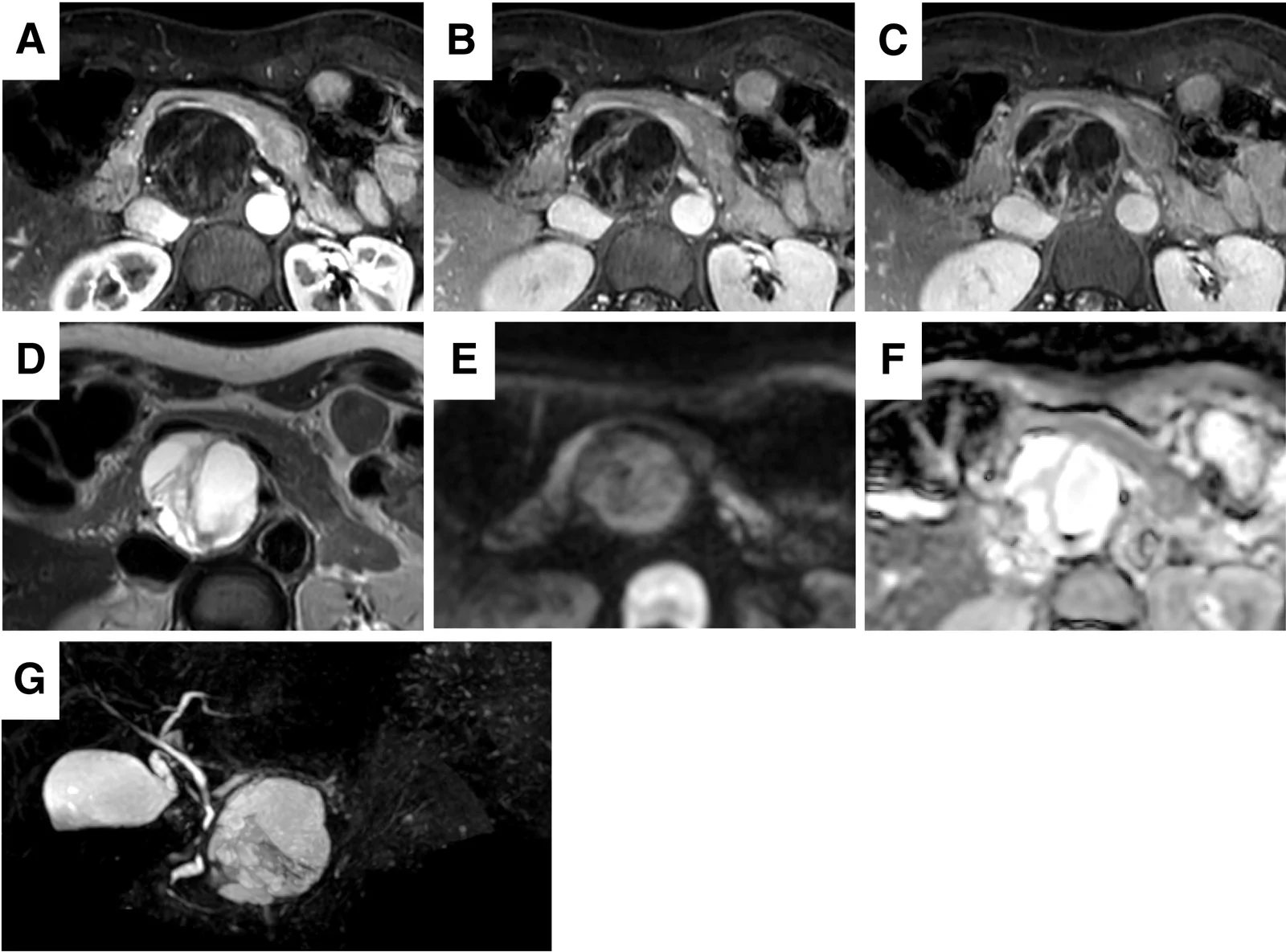
MRI and MRCP findings. (**A**–**C**) Dynamic contrast-enhanced, fat-suppressed T1-weighted images obtained during the arterial (**A**), portal venous (**B**), and equilibrium (**C**) phases showing gradual enhancement of the septa and inner cyst wall. (**D**) T2-weighted image showing a multilocular cystic lesion dorsal to the pancreatic head, with locules of various sizes separated by irregular septa. (**E**) Diffusion-weighted image showing relatively high signal intensity in the thickened septal portions. (**F**) ADC map showing no definite diffusion restriction throughout the lesion. (**G**) MRCP showing no obvious communication between the lesion and the main pancreatic duct. ADC, apparent diffusion coefficient; MRCP, magnetic resonance cholangiopancreatography

MRI demonstrated a multilocular cystic lesion dorsal to the pancreatic head (**Fig. 2**). Dynamic contrast-enhanced, fat-suppressed T1-weighted images showed gradual enhancement of the septa and inner cyst wall (**Fig. 2A**–**2C**). T2-weighted images showed a multilocular cystic architecture with locules of various sizes separated by irregular septa (**Fig. 2D**). Diffusion-weighted images showed relatively high signal intensity in the thickened septal portions (**Fig. 2E**), whereas the ADC map showed no definite diffusion restriction throughout the lesion (**Fig. 2F**). MRCP showed no obvious communication between the lesion and the main pancreatic duct (**Fig. 2G**). These imaging findings further supported the preoperative diagnosis of an MCN-like cystic neoplasm in the pancreatic head region.

**Fig. 3:**
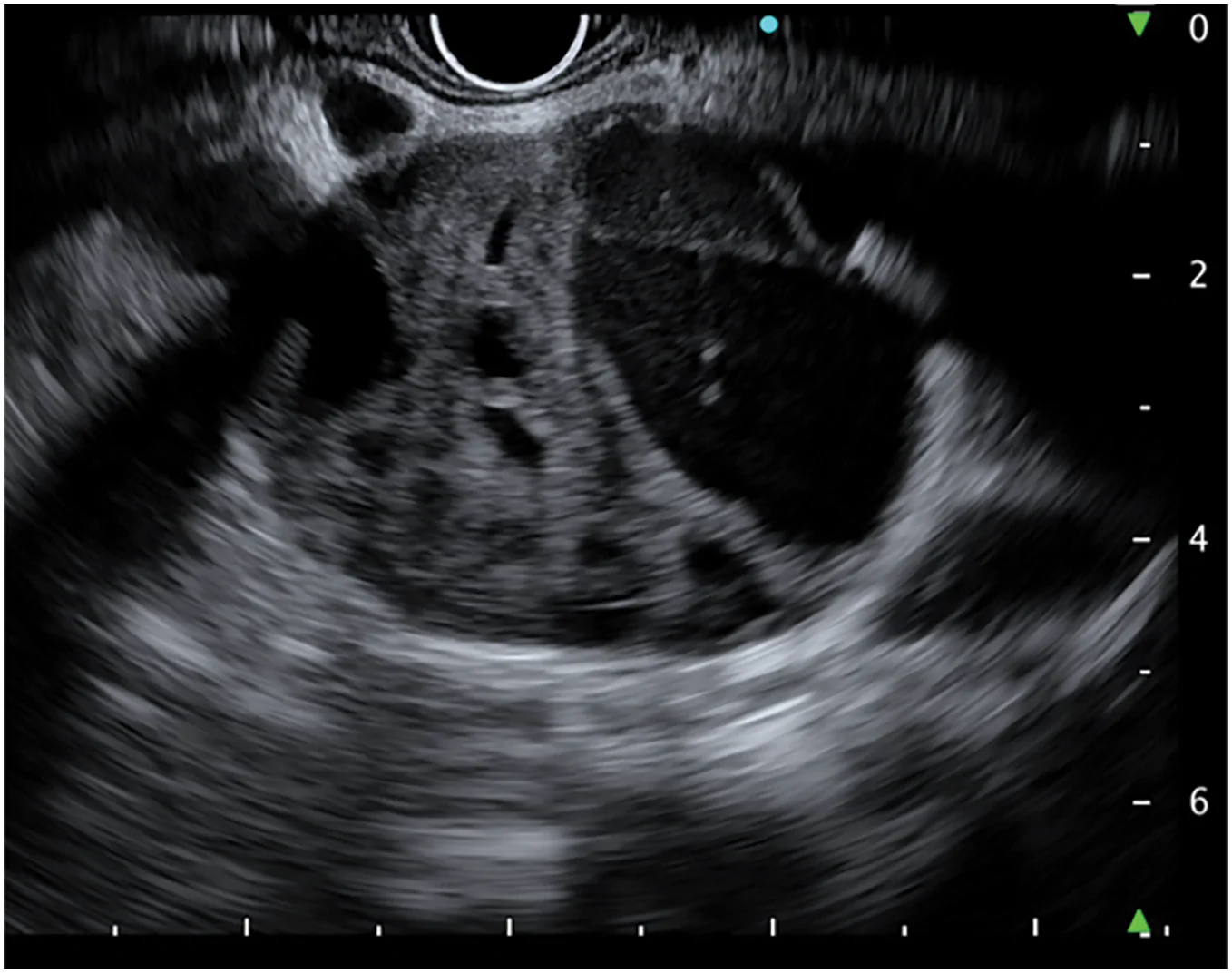
EUS findings. EUS showing a well-defined cystic lesion measuring approximately 5 cm dorsal to the pancreatic head region. The lesion has a thickened wall and heterogeneous internal architecture consisting of multiple cystic spaces and coarse solid components. Although most of the lesion appears to be located outside the pancreatic parenchyma and compresses the pancreatic head from the dorsal aspect, an exophytic tumor arising from the uncinate process could not be excluded. EUS, endoscopic ultrasonography

EUS revealed a well-defined cystic lesion measuring approximately 5 cm dorsal to the pancreatic head region (**Fig. 3**). The lesion had a thickened wall and heterogeneous internal architecture consisting of multiple cystic spaces and coarse solid components. Although most of the lesion appeared to be located outside the pancreatic parenchyma, an exophytic tumor arising from the uncinate process could not be excluded. The differential diagnosis included an MCN-like cystic neoplasm, a solid-pseudopapillary neoplasm, a cystic neuroendocrine tumor, a GIST, a neurogenic tumor, and other pancreatic malignancies. EUS targeting the solid or extrapancreatic component might have provided diagnostic tissues. However, it was not performed because the lesion was regarded at the time as a cystic pancreatic neoplasm with malignant potential, and puncture-related dissemination was a concern. In retrospect, such targeted sampling could have been considered because the result might have influenced the treatment strategy. FDG-PET/CT was not performed.

Surgical exploration revealed a whitish, elastic-firm cystic mass measuring approximately 5 cm in the pancreatic head region. During Kocher mobilization, the tumor was mobilized together with the pancreatic head, making a primary retroperitoneal origin less likely. Because the working diagnosis remained an MCN-like cystic neoplasm with malignant potential, malignancy could not be excluded; moreover, safe local excision was considered technically unsafe given the tumor location and close vascular relationships. Therefore, subtotal stomach-preserving pancreaticoduodenectomy with lymphadenectomy was selected.

**Fig. 4:**
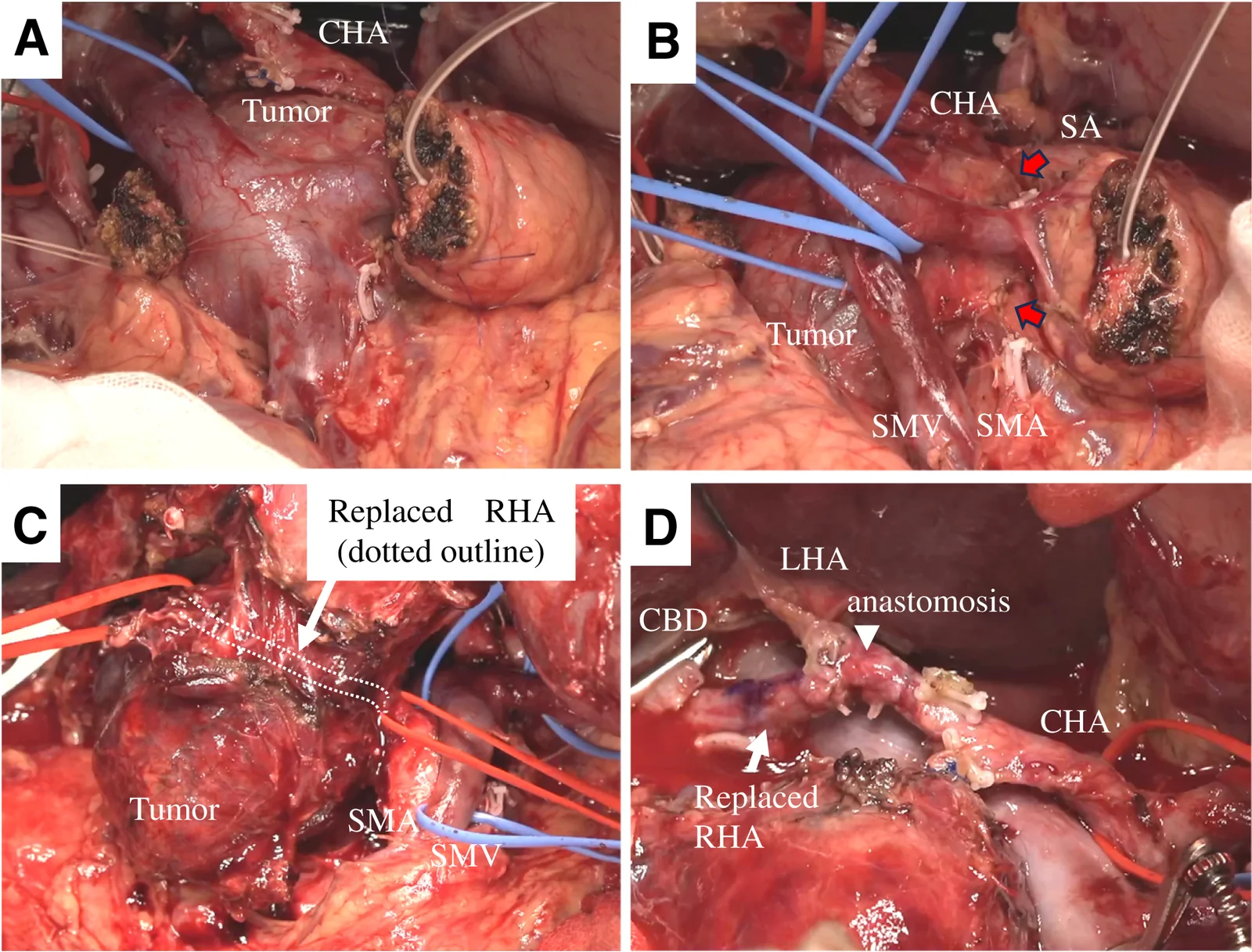
Intraoperative findings. (**A**) Intraoperative view after pancreatic transection showing the tumor compressing the porto-mesenteric venous axis anteriorly. (**B**) Intraoperative view after dissection of the porto-mesenteric venous axis showing firm adhesion of the tumor to the periarterial nerve plexus around the CHA, SA, and SMA (red arrow). (**C**) Intraoperative view showing the replaced RHA coursing immediately adjacent to the tumor. The dotted line traces the artery along the tumor’s surface. The artery was encircled with red vessel loops at its origin from the SMA and on the hepatic side. (**D**) Intraoperative photograph after arterial reconstruction showing end-to-side anastomosis of the replaced RHA to the LHA. Anastomotic patency and intrahepatic arterial flow in the right hepatic lobe were confirmed by intraoperative Doppler ultrasonography. CBD, common bile duct; CHA, common hepatic artery; LHA, left hepatic artery; RHA, right hepatic artery; SA, splenic artery; SMA, superior mesenteric artery; SMV, superior mesenteric vein

After pancreatic transection, the tumor was found to compress the portal vein, SMV, and splenic vein anteriorly. However, the tumor could be separated from the porto-mesenteric venous axis because only fibrous adhesions were present, and venous resection was not required (**Fig. 4A**). In contrast, the tumor was firmly adherent to the periarterial nerve plexus surrounding the CHA, celiac axis, SA, and SMA, particularly around the celiac axis and SMA root (**Fig. 4B**). The replaced RHA arose from the SMA and coursed immediately adjacent to the tumor, with broad contact over approximately 5 cm (**Fig. 4C**). Although preoperative imaging showed no definite arterial invasion, dense fibrous adhesion extended along the artery, and no safe adventitial dissection plane could be identified. Because malignancy remained possible and forced separation carried risks of arterial injury, capsular rupture, and incomplete resection, the replaced RHA was resected en bloc with the tumor. Clamp testing demonstrated loss of arterial flow to the right hepatic lobe; therefore, the replaced RHA was reconstructed by end-to-side anastomosis to the LHA using 8-0 polypropylene sutures (8-0 double-armed PRONOVA suture; Ethicon, Inc., Somerville, NJ, USA) (**Fig. 4D**). After reconstruction, anastomotic patency and intrahepatic arterial flow in the right hepatic lobe were confirmed by intraoperative Doppler ultrasonography. Indocyanine green (ICG) fluorescence imaging was not used, and no separate dedicated assessment of biliary perfusion was performed. The tumor was removed en bloc without capsular rupture. The operation time was 567 min, and the estimated blood loss was 480 mL. No blood transfusion was required.

**Fig. 5:**
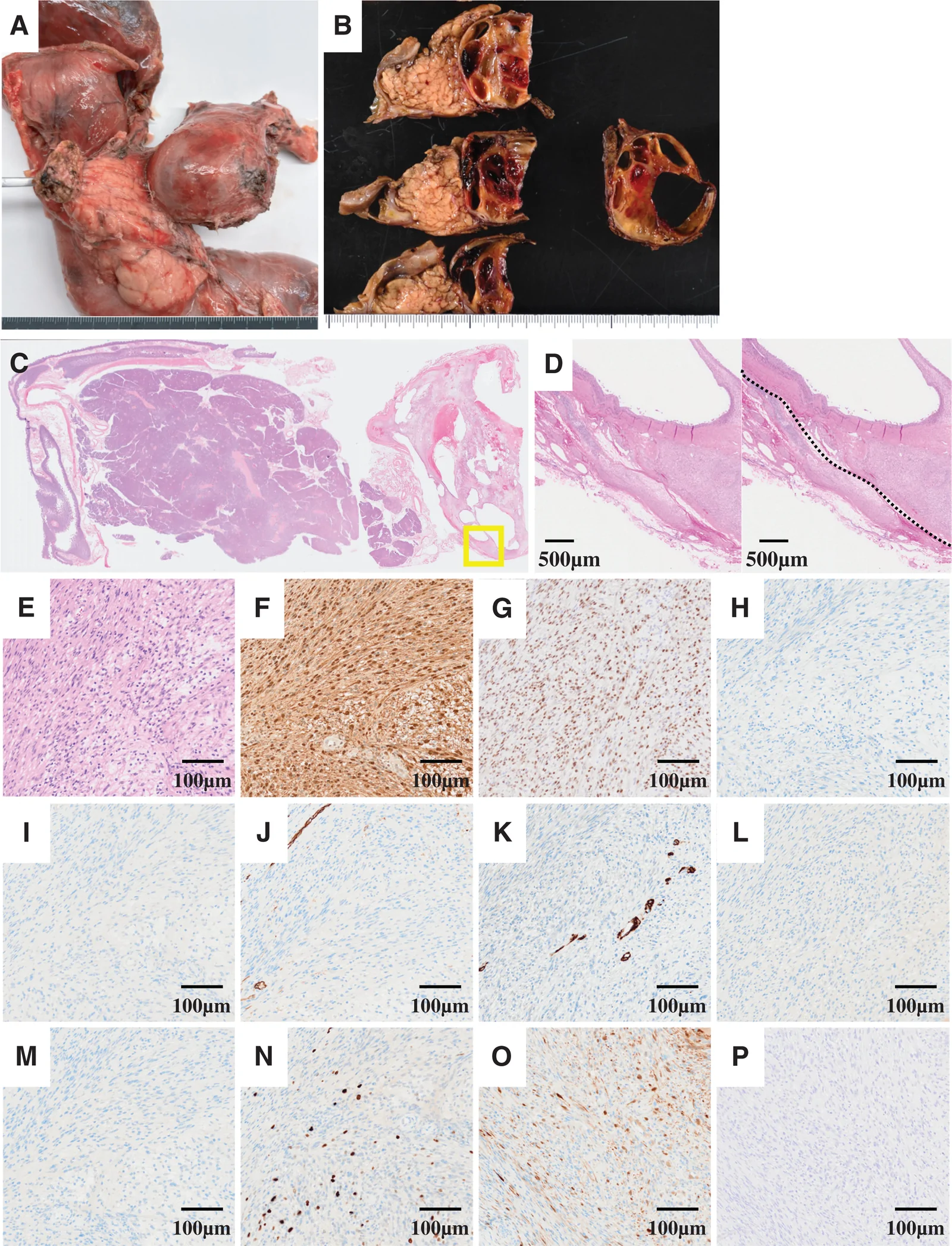
Macroscopic and histopathological findings of the resected tumor. (**A**) Gross appearance of the resected specimen showing a well-circumscribed, elastic-firm tumor measuring approximately 5 cm, located on the posterior aspect of the pancreatic head, corresponding to the pancreatic head nerve plexus region. Nerve plexus tissue is present around the tumor. (**B**) Serial sections of the tumor showing multilocular cystic spaces, prominent intratumoral hemorrhage, and thick fibrous septa. (**C**) Low-power hematoxylin and eosin staining showing a relatively well-circumscribed spindle-cell neoplasm with extensive cystic and hemorrhagic degeneration. The tumor is clearly demarcated from the adjacent pancreatic parenchyma. (**D**) Low-power magnification of the yellow boxed area in (**C**). The tumor is adjacent to surrounding normal pancreatic head nerve plexus tissue, and the interface is indicated by a black dotted line in the right-hand image. (**E**) High-power hematoxylin and eosin staining showing round-to-spindle-shaped tumor cells arranged in fascicular, microcystic, and haphazard patterns. (**F**, **G**) Immunohistochemical staining showing diffuse positivity for S-100 protein (**F**) and SOX10 (**G**). (**H**–**M**) Immunohistochemical staining showing negativity for c-kit (**H**), DOG1 (**I**), CD34 (**J**), α-SMA (**K**), desmin (**L**), and AE1/AE3 (**M**). (**N**) Ki-67 immunostaining showing a labeling index of 11.7%. (**O**, **P**) Immunohistochemical staining showing retained p16 expression (**O**) and negativity for EGFR (**P**). Scale bars: 500 μm in (**D**) and 100 μm in (**E**–**P**). AE1/AE3, cytokeratin AE1/AE3; CD34, cluster of differentiation 34; DOG1, discovered on gastrointestinal stromal tumor 1; EGFR, epidermal growth factor receptor; SOX10, SRY-box transcription factor 10; α-SMA, alpha-smooth muscle actin

Gross examination of the resected specimen showed a well-circumscribed, elastic-firm tumor measuring approximately 5 cm on the posterior aspect of the pancreatic head, corresponding to the region of the pancreatic head nerve plexus region (**Fig. 5A**). Serial sections showed multilocular cystic spaces with prominent intratumoral hemorrhage and thick fibrous septa (**Fig. 5B**).

Histopathological examination revealed a relatively well-circumscribed spindle-cell neoplasm with extensive cystic and hemorrhagic degeneration (**Fig. 5C**). The tumor was clearly demarcated from the adjacent pancreatic parenchyma, and nerve plexus tissue corresponding to the pancreatic head nerve plexus was observed around the tumor (**Fig. 5D**). These findings supported the interpretation that the tumor was located in the pancreatic head nerve plexus region rather than arising from the pancreatic parenchyma itself. No epithelial lining was identified within the cystic spaces. On higher magnification, round to spindle-shaped tumor cells were arranged in fascicular, microcystic, and haphazard patterns, without marked cytological atypia, mitotic figures, coagulative tumor necrosis, or infiltrative growth (**Fig. 5E**). Immunohistochemically, the tumor cells showed diffuse positivity for S-100 protein and SRY-box transcription factor 10 (SOX10) (**Fig. 5F**, **5G**), supporting Schwann-cell differentiation. The tumor cells were negative for c-kit, discovered on gastrointestinal stromal tumor 1 (DOG1), CD34, α-SMA, desmin, and pan-cytokeratin AE1/AE3 (AE1/AE3) (**Fig. 5H**–**5M**). The Ki-67 labeling index was 11.7% (**Fig. 5N**). Although this index was relatively high, the overall morphology and immunophenotype, including retained p16 expression and absence of EGFR expression (**Fig. 5O**, **5P**), did not support a malignant peripheral nerve sheath tumor. Surgical margins were negative. Taken together, the lesion was diagnosed as a schwannoma likely arising from the pancreatic head nerve plexus.

The postoperative course was uneventful, and the patient was discharged on POD 20. Dynamic CT performed 1 week after surgery confirmed the patency of the reconstructed artery. Follow-up CT at 3 months showed maintained arterial patency and no evidence of recurrence. At 4 months after surgery, the patient remained clinically well with no evidence of recurrence. Given the short follow-up period and the relatively high Ki-67 labeling index, continued long-term clinical and radiological surveillance was planned.

## DISCUSSION

Pancreatic and peripancreatic schwannomas are exceptionally rare, and preoperative diagnosis remains challenging. Previous reviews have shown that these tumors may mimic a wide spectrum of pancreatic lesions, including MCN, serous cystic neoplasm, solid-pseudopapillary neoplasm, neuroendocrine tumor, and pseudocyst.^1–3)^ This diagnostic difficulty reflects the intrinsic histopathological heterogeneity of schwannomas. Schwannomas are typically composed of hypercellular Antoni A areas and hypocellular Antoni B areas, the latter of which are prone to degenerative changes such as cyst formation, hemorrhage, hyalinization, calcification, and necrosis.^1,2,7)^ Thus, the mixed solid and cystic appearance of a pancreatic schwannoma represents the radiologic expression of its underlying histologic architecture.

This radiologic–pathologic correlation explains the findings in the present case. The thickened enhancing components may have corresponded to cellular Antoni A areas, whereas the multilocular cystic spaces and hemorrhagic components likely reflected degenerative changes within Antoni B-rich areas. MRI studies have reported that pancreatic schwannomas often show T1 hypointensity, heterogeneous T2 hyperintensity, high signal intensity on diffusion-weighted imaging, and gradual or delayed enhancement.^8)^ Although these findings may provide diagnostic clues, they are not sufficiently specific to establish the diagnosis, particularly when the lesion presents as a multilocular cystic tumor in the pancreatic head region.

Recent reports further illustrate this diagnostic challenge. Jemaneh et al. reported a pancreatic schwannoma presenting as a cystic pancreatic mass that was treated with a Whipple procedure, and Gupta et al. described a pancreatic head schwannoma that clinically and radiologically resembled a malignant pancreatic head tumor and underwent pancreaticoduodenectomy before the histological diagnosis was established.^9,10)^ Conversely, when a schwannoma is suspected and a tissue diagnosis is obtained preoperatively, less invasive management may be possible. Hanaoka et al. reported a pancreatic schwannoma diagnosed by EUS-FNA, in which surgery was avoided because the diagnosis was established before treatment.^11)^ Kato et al. also reported a pancreatic schwannoma diagnosed by EUS-guided tissue acquisition and managed without resection.^12)^ In addition, Alvarez et al. described a case in which EUS-FNB with immunohistochemical confirmation of S-100 positivity enabled a preoperative diagnosis of pancreatic schwannoma, followed by laparoscopic enucleation.^13)^ These reports indicate that including a schwannoma in the differential diagnosis may expand treatment options, including observation, enucleation, local excision, or other organ-preserving procedures in carefully selected patients.

In this context, EUS-guided tissue acquisition may aid the diagnosis and management of a pancreatic schwannoma. Because imaging alone is often insufficient to distinguish a pancreatic schwannoma from other pancreatic neoplasms, sampling of a solid or thickened component can provide material for cytological or histological evaluation and immunohistochemical confirmation of Schwann-cell differentiation, including S-100 protein and SOX10 expression.^12,14)^ Takasumi et al. reported that EUS-FNA contributed to the diagnosis of schwannoma in selected cases, although inadequate sampling and insufficient material for immunohistochemistry remained important limitations.^15)^ However, the indication for EUS-guided tissue acquisition in pancreatic cystic lesions should be determined cautiously and on a case-by-case basis. European evidence-based guidelines recommend considering EUS-FNA when CT or MRI findings are inconclusive and when the results are expected to influence management.^16)^ Although needle-tract seeding or tumor dissemination after EUS-guided tissue acquisition is rare, such events have been reported in pancreatic lesions, including cystic and IPMN-related lesions.^17,18)^ Therefore, when an MCN-like cystic neoplasm or another cystic tumor with malignant potential is suspected, the indication for EUS-guided tissue acquisition should be individualized by weighing the potential diagnostic benefit against the risk of puncture-related dissemination. In the present case, tissue acquisition was not performed because the lesion was considered an MCN-like cystic neoplasm with malignant potential. However, given the predominantly extrapancreatic location of the lesion and the presence of a safely accessible solid component, targeted EUS-guided tissue acquisition rather than cyst-fluid aspiration could have provided additional diagnostic information and potentially altered the surgical strategy. The lack of preoperative tissue acquisition therefore represents a limitation in the diagnostic evaluation and therapeutic decision-making in this case.

FDG-PET/CT was not performed in this case. Its absence limited metabolic characterization and whole-body staging. However, FDG uptake in benign schwannomas is highly variable and may be intense; consequently, PET/CT cannot reliably distinguish benign schwannomas from malignant peripheral nerve sheath tumors or other malignancies.^19)^ PET/CT might therefore have supplemented, but would not necessarily have resolved, the preoperative diagnostic uncertainty.

The exact anatomical origin should be described cautiously. Operative and pathological findings showed that the tumor was demarcated from the pancreatic parenchyma and surrounded by tissue of the pancreatic head nerve plexus, supporting the interpretation that it was located in this region and likely arose from it. However, direct continuity with a specific nerve was not demonstrated. Accordingly, we describe the tumor as “likely arising from the pancreatic head nerve plexus” or “located in the region of the pancreatic head nerve plexus” rather than making a definitive claim regarding its origin.^4,5)^

This distinction has important surgical implications. When a schwannoma is diagnosed preoperatively and can be safely separated from adjacent structures, limited excision or organ-preserving surgery may be appropriate.^4,6)^ In the present case, pancreaticoduodenectomy was selected because malignancy could not be excluded and local excision was considered technically unsafe. Although the tumor was separable from the porto-mesenteric venous axis, it was firmly adherent to the periarterial nerve plexus around the celiac axis, CHA, SA, and SMA. A replaced RHA arising from the SMA was also in broad contact with the tumor. Because adequate exposure and safe separation were not feasible without pancreatic transection, local excision was considered to carry substantial risks of vascular injury, capsular rupture, and incomplete resection. Pancreaticoduodenectomy with en bloc tumor resection and reconstruction of the replaced RHA was therefore considered the safest strategy under these intraoperative circumstances.

In retrospect, the marked arterial displacement and compression without definite radiological signs of invasion may have reflected the schwannoma’s close attachment to the periarterial nerve plexus. In similar cases, such findings may alert surgeons to the possibility of dense periarterial adhesion and technically challenging tumor separation.

The pathological findings favored a benign schwannoma despite a relatively high Ki-67 labeling index of 11.7%. The tumor was well circumscribed and lacked marked cytological atypia, mitotic figures, coagulative necrosis, or infiltrative growth. Diffuse expression of S-100 and SOX10 supported Schwann cell differentiation. In contrast, negativity for c-kit, DOG1, and CD34 argued against the GIST, whereas negativity for desmin and α-SMA did not support smooth muscle or myofibroblastic differentiation.^2,20)^ The absence of AE1/AE3 expression also did not support an epithelial neoplasm, including pancreatic sarcomatoid carcinoma.^21)^ Nevertheless, these findings should be interpreted as part of the overall immunohistochemical profile because aberrant keratin expression has occasionally been reported in schwannomas, particularly those arising in the retroperitoneum.^22,23)^

Although the Ki-67 labeling index was relatively elevated, no mitotic figures or overt malignant morphological features were identified. Retained p16 expression and the absence of EGFR expression also did not support a malignant peripheral nerve sheath tumor, which more commonly exhibits loss of p16, EGFR expression, reduced or heterogeneous Schwann cell marker expression, and greater proliferative activity.^14,24)^ Taken together, the morphological and immunohistochemical findings supported the diagnosis of schwannomas. Combined with the operative and topographic findings, they also supported a likely origin in the pancreatic head nerve plexus.

## CONCLUSIONS

A schwannoma located in the region of the pancreatic head nerve plexus may closely mimic an MCN-like pancreatic cystic neoplasm because of cystic and hemorrhagic degeneration. When clinically appropriate and safely feasible, EUS-guided tissue acquisition may be considered, as an accurate preoperative diagnosis may permit observation or organ-preserving surgery. However, when marked compression or displacement of major vessels is accompanied by firm periarterial adhesion that precludes safe tumor separation, the potential need for pancreaticoduodenectomy should be anticipated to achieve safe and complete tumor removal.

## DECLARATIONS

### Use of generative artificial intelligence

During the preparation of this manuscript, the authors used ChatGPT (OpenAI) solely for English-language editing to improve grammar, wording, and readability. The tool was not used for study design, data collection or analysis, interpretation of the findings, or the creation or modification of figures. All AI-assisted revisions were reviewed and verified by the authors, who take full responsibility for the content of the manuscript.

### Availability of data and materials

The data and materials used in this case report are not publicly available due to patient privacy concerns.
